# Effect of Sublethal Concentrations of Metal Nanomaterials on Cell Energy Metabolism

**DOI:** 10.3390/toxics11050453

**Published:** 2023-05-11

**Authors:** Chaoshuai Liang, Qiuyao Jiang, Zhenzhen Liu, Jian Yang, Jie Zhang, Shuping Zhang, Wei Xin

**Affiliations:** 1 Shandong Provincial Hospital Affiliated to Shandong First Medical University, Jinan 250000, China; lcszx1@yeah.net; 2 Medical Science and Technology Innovation Center, Shandong First Medical University, Jinan 250000, China; qiuyao8483@163.com (Q.J.);

**Keywords:** metallic nanomaterials, mitochondria damage, energy metabolism

## Abstract

Metallic nanomaterials (MNMs) are widely used in the medical field because of their photocatalytic, optical, electrical, electronic, antibacterial, and bactericidal properties. Despite the advantages of MNMs, there is a lack of complete understanding of their toxicological behavior and their interactions with cellular mechanisms that determine cell fate. Most of the existing studies are acute toxicity studies with high doses, which is not conducive to understanding the toxic effects and mechanisms of homeostasis-dependent organelles, such as mitochondria, which are involved in many cellular functions. In this study, four types of MNMs were used to investigate the effects of metallic nanomaterials on mitochondrial function and structure. We first characterized the four MNMs and selected the appropriate sublethal concentration for application in cells. Mitochondrial characterization, energy metabolism, mitochondrial damage, mitochondrial complex activity, and expression levels were evaluated using various biological methods. The results showed that the four types of MNMs greatly inhibited mitochondrial function and cell energy metabolism and that the material entering the mitochondria damaged the mitochondrial structure. Additionally, the complex activity of mitochondrial electron transport chains is critical for assessing the mitochondrial toxicity of MNMs, which may serve as an early warning of MNM-induced mitochondrial dysfunction and cytotoxicity.

## 1. Introduction

In the cutting-edge scientific world, nanomaterials (NMs) have attracted the attention of scientists because they have been used in biomedical and chemical industries, drug delivery, electronics, and environmental remediation based on their properties, such as size, distribution, and morphology [1,2]. NMs are classified according to their state, chemical composition, shape, size, and dimensions and include carbon-based NMs, metal-based NMs, dendrimers, and nanocomposites [3]. Among them, metal-based NMs are considered one of the most promising systems owing to they have largest output and have been used in biomedical field. In biomedical field, metal-based NMs can be prepared and modified to conjugate with drugs or ligands [4,5]. They also can be made as biosensors, bioimaging, hyperthermia, photoablation, and tumor targeting [6,7]. However, with the increasing use and production of metal-based NMs, occupational exposure to them is also increasing. Concerns have been raised about the balance between the environment and the ecosystem, which has become a driving force for scientists, who are increasingly motivated to investigate the effects of metal-based NMs on health and the difficulties in determining their toxicity. Although some studies have focused on toxicity assessments of metal-based NMs [8,9], there was an incomplete understanding of their toxicological behavior and their interactions with cellular mechanisms that determine cell fate [10].

ZnO, CuO, and Ag are the most widely used metal-based NMs, used in almost every field of life. They are synthesized by chemical and physical approaches, and their wide application is due to their unique characteristics, such as photocatalysis, optics, electricity, electronics, and antibacterial properties [11,12]. However, how to safely use these nanomaterials and whether they have long-term or short-term health effects also require attention. The toxicity of metal-based NMs to health occurs through inhalation, ingestion, and injection as they accumulate in organisms [1,13]. Furthermore, the toxicity of metal-based NMs depends on their dose and shape [14]. To assess the toxicity of metal-based NMs in the body, the toxic effects of different concentrations, sizes, and shapes of MNMs can be studied using various biological and medical approaches.

A study using three experimental rat models evaluated the therapeutic effect of ZnO nanomaterials at different doses, resulting in continuous increases in serum creatinine and urea nitrogen levels, impairment of renal function, and renal toxicity [15]. ZnO and CuO of various sizes and shapes were selected to compare the toxicity of the air–blood barrier permeability using an in vitro model [16]. It was found that different sizes of ZnO and CuO had varying permeability effects, causing the air–blood barrier system to become more permeable. This causes more pollutants and exogenous inflammatory factors to circulate within organisms, damaging organs. Previous studies have reported that metal-based NMs are hazardous to cells and organisms [17]. ZnO nanomaterials exhibited a dose-dependent effect on the survival rate of the human hepatoma cell line HepG2 with 29.1 μg/mL of IC_50_ for 90 nm ZnO nanomaterials [17]. Similarly, the adverse effect of CuO nanomaterials on HEK cells was also dose-dependent with 65 μg/mL of IC_50_ [18]. In addition, Ag nanomaterials can promote wound healing; however, they can cause cell damage [19]. These and other studies have shown that metal-based NMs are hazardous to cells and organisms [20,21,22]. For Ag nanomaterials, endothelial cell line HBEC5i showed obvious membrane damage 24 h after the treatment of Ag nanoparticles in a dose-dependent way. Moreover, the IC_50_ of 25 nm Ag nanomaterials in macrophages was 25 μg/mL [23]. The apoptosis rate of macrophages was significantly increased after 20 μg/mL AgNWs exposure [24]. However, most of the current studies have focused on the cytotoxic effects of metal-based NMs [25,26,27], while the damage induced by these metal-based NMs at sublethal levels has received less attention. More importantly, it is still unclear whether the sublethal concentrations of these nanomaterials affect the structure and function of organelles. In this study, we hypothesized that sublethal concentrations of ZnO, CuO, Ag, and AgNMs did not cause cell death. To confirm this hypothesis, we selected renal tubular epithelial cells (HK-2 cells) as a cell model because the kidneys are an important target for potential harm from NMs, as waste and toxic substances are usually excreted in the urine [28]. Through CCK8 and flow cytometry analyses, 10 μg/mL of NMs was determined to be the sublethal concentration. Surprisingly, cell energy metabolism analysis confirmed that this sublethal concentration caused mitochondrial damage. 

## 2. Materials and Methods

### 2.1. Morphological and Surface Characterization of the Four Metallic Nanomaterials

Thirty of microliters of ZnO, CuO, Ag, and AgNW NM suspensions (100 µg/mL) in ethanol were dropped onto a carbon-coated 200-mesh copper grid (Zhongjingkeyi Technology, Beijing, China). The grids were observed using transmission electron microscopy (TEM; HT7800, HITACHI, Tokyo, Japan) operating at an acceleration voltage of 120 kV and equipped with a complementary metal oxide semiconductor (CMOS) digital camera.

The hydrodynamic size distribution of the ZnO, CuO, Ag, and AgNW nanomaterials was evaluated by dynamic light scattering (DLS) analysis using a Malvern Zetasizer Pro (Malvern Zetasizer Pro, Worcestershire, UK). The hydrodynamic behavior was assessed by dispersing these NMs (final concentration, 100 µg/mL) in Milli-Q (mQ) water and medium. At least three biological replicates were examined for each experiment.

The zeta potential distribution of the NMs was evaluated by electrophoretic light scattering (ELS) using a Malvern Zetasizer Pro. The hydrodynamic behavior was assessed by dispersing these nanomaterials (final concentration, 100 µg/mL) in Milli-Q (mQ) water. At least three biological replicates were examined for each experiment.

### 2.2. Cell Culture

Human proximal tubule epithelial cells (HK-2) were derived from a US-type culture set (Rockville, MD, USA). The cells were cultured in RPMI 1640 medium (Gibco™, Grand Island, NY, USA) containing 10% fetal bovine serum (FBS, Gibco^®^, Grand Island, NY, USA) and 1% penicillin-streptomycin at 37 °C with 5% CO_2_ for 24 h. Then, 5, 10, 20, and 30 μg/mL of ZnO, CuO, Ag, and AgNW were treated with HK-2 cells for 24 h. The treated HK-2 cells were used for subsequent experiments.

### 2.3. Cell Viability

HK-2 cell viability was assessed using a Cell Counting Kit 8 (CCK8; MCE, Romulus, MI, USA). Briefly, the cell medium was removed, and replaced with a fresh RPMI 1640 medium, 10 μL of CCK8 solution was added to each well of the 96-well plate containing treated and untreated HK-2 cells, and cells were incubated for 1 h at 37 °C. Plates were analyzed using a Varioskan LUX plate reader (Thermo Fisher Scientific, Waltham, MA, USA). At least three biological replicates were examined for each experiment.

### 2.4. Apoptosis Detection by Flow Cytometry

HK-2 cells were treated with NMs for 24 h. The cells were collected and washed twice with PBS. Thereafter, cells were stained with 5 μL of Annexin-V FITC (BD Biosciences, San Jose, CA, USA) and 5 μL of PI (BD Biosciences, San Jose, CA, USA) for 15 min following the instructions from the manufacturer, followed by flow cytometry analysis by ATTUNE NXT (Invitrogen, Waltham, MA, USA). At least three biological replicates were examined for each experiment.

### 2.5. Real-Time RT-PCR Analysis

Total RNA was extracted from the treated HK-2 cells using TRIzol Reagent (Invitrogen, No. 15596026). mRNA was reversed transcribed into cDNA with the PrimeScript RT reagent kit (Takara, Tokyo, Japan) in a 20 μL reaction system. The qRT-PCRs were carried out on a Roche LightCycle 480 (Roche Applied Sciences, Mannheim, Germany) under the following thermal cycling conditions: 95 °C for 30 s, 40 cycles of 95 °C for 5 s, and 60 °C for 30 s. Each targeting gene expression was normalized with GAPDH. The primers used in this study are listed in Table 1. At least three biological replicates were examined for each experiment.

### 2.6. Western Blot Analysis

Cells were collected from a six-well plate and cleaved in RIPA lysis buffer containing a cocktail of protease and phosphatase inhibitors (Solarbio, Beijing, China). The proteins were isolated and transferred to a PVDF membrane (Millipore, Billerica, MA, USA) using 12% SDS-PAGE. Plug the membrane with 5% skim milk at room temperature (RT) for 1 h. The membranes were then incubated with the following primary antibodies: Total OXPHOS Human WB Antibody Cocktail (1:1000, Abcam, Cambridge, UK), GAPDH mouse mAb (1:5000, ProteinTech Group, Wuhan, China), β-actin (1:4000, ProteinTech Group, Wuhan, China), and Tubulin (1:4000, ProteinTech Group, Wuhan, China) and were then incubated overnight with the primary antibody (Abcam, Cambridge, UK) at 4 °C. An enzyme-labeled goat anti-mouse secondary antibody (1:5000; ProteinTech Group, Wuhan, China) was used to detect the primary antibody and ChemiDoc XRS+ (Bio-Rad, Hercules, CA, USA) was used for imaging. Image Lab Software 3.0 (Bio-Rad, Hercules, CA, USA) was used to analyze the images. At least three biological replicates were examined for each experiment.

### 2.7. Morphological Changes in Mitochondria Were Observed by TEM

Ultrathin sections of the cells were examined using TEM to reveal the morphology of the mitochondria. The cells were then treated with 10 μg/mL of the NM for 24 h and washed three times with PBS. 

### 2.8. Measurement of Mitochondrial Membrane Potential

Mitochondrial membrane potential was measured using JC-1 dye (Invitrogen, USA). The two colors monitored were green and red at 529 and 590 nm, respectively. Cells were seeded in confocal dishes (Nest, Wuxi, China). After 24 h of treatment, the cells were placed in a fresh medium containing JC-1 (final concentration was 1.5 μM) and cultured at 37 °C for 20 min. After cleaning three times, a Cell Discoverer 7 (ZEISS, Oberkochen, Germany) analysis was performed.

### 2.9. Mitochondrial Energy Metabolism Analysis System

The cell oxygen consumption rate (OCR) was measured using an Agilent Seahorse XF Cell Mitochondrial Stress Test kit (103015-100) to characterize the key parameters of mitochondrial function. HK-2 cells in the culture plate were treated with 10 μg/mL of NMs and cultured for 24 h. Subsequently, the user guide for the experiment was followed. Using a Seahorse XFe/XF analyzer (Agilent, Santa Clara, CA, USA), the data were analyzed using the Seahorse XF Mitochondrial Stress Test Report Generator. At least three biological replicates were examined for each experiment.

### 2.10. Measurement of Mitochondrial Complex Activity

HK-2 cells (5 × 10^6^) were collected from treated and untreated groups. The cells were analyzed according to the instructions of the mitochondrial respiratory chain complex (I–V) kit (Solarbio) and detected using Varioskan LUX (Thermo Fisher Scientific, Skanlt Software 6.1, Waltham, MA, USA). At least three biological replicates were examined for each experiment.

### 2.11. Statistical Analysis

Data were reported as means and standard error of the mean (SEM) using GraphPad Prism 9.2.0.332 (GraphPad Software). To determine the statistical significance between multiple groups, one-way analysis of variance (ANOVA), followed by Bonferroni’s post hoc test, was performed using GraphPad Prism. Statistical significance was set at *p* < 0.05.

## 3. Results and Discussion

### 3.1. Characterization of Metallic Nanomaterials

**Figure 1:**
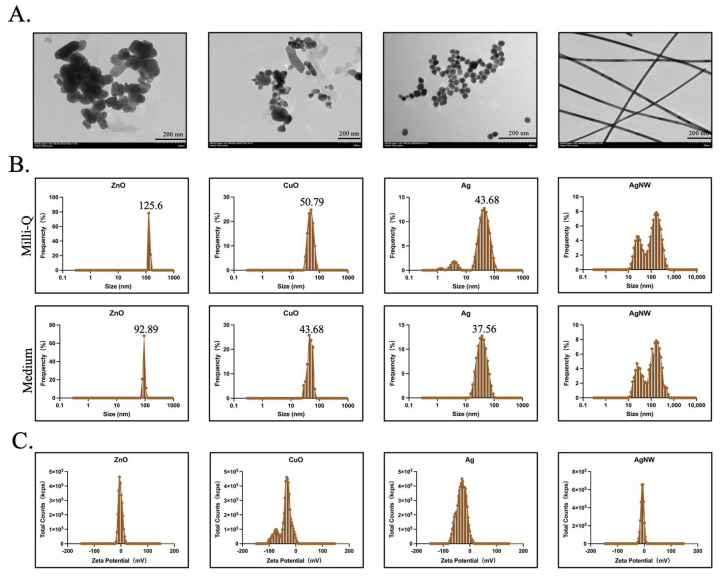
Characterization of the four types of metallic nanomaterials. (**A**) Representative TEM image of the four types of metallic nanomaterials used in this study. Scale bar is 200 nm. (**B**) The hydrodynamic size distribution of ZnO, CuO, Ag, and AgNW was evaluated by DLS. (**C**) The zeta potential distribution of ZnO, CuO, Ag, and AgNW was evaluated by ELS.

In this study, the four typical metallic nanomaterials (ZnO, CuO, Ag, and AgNW) were selected because of their typical cytotoxicity [15,16,29], high biocompatibility [30], and applications in various fields, such as biological and medical systems [31,32,33]. TEM imaging demonstrated that ZnO, CuO, and Ag were spherical. A portion of the ZnO was rod-shaped and the AgNWs were long wires (Figure 1A). DLS was used to measure the size of nanomaterials under the Milli-Q water and cell medium. The average sizes of ZnO, CuO, Ag, and AgNW were 129.5 ± 38.7 nm (*n* = 3), 50.1 ± 10.9 nm (*n* = 3), 30.3 ± 0.4 nm (*n* = 3), and 245.1 ± 1.5 nm (*n* = 3) in diameter under Milli-Q water, respectively. Under cell medium, the average sizes of ZnO, CuO, Ag, and AgNW were 92.89 ± 13.4 nm (*n* = 3), 43.68 ± 9.4 nm (*n* = 3), 37.56 ± 0.8 nm (*n* = 3), and 243.5 ± 2.1 nm (*n* = 3) in diameter, respectively (Figure 1B). Apart from the AgNWs, the particle sizes of the other three NMs were consistent with those provided by the supplier (XFNANO, Nanjing, China) (Table 2). AgNWs exhibited a larger size than that presented in Table 2, and we hypothesized that this larger value was due to the length of the AgNM. We measured the diameter of AgNW by ImageJ (version 2.9.0) based on the TEM image of AgNW in Figure 1A and the average diameter was found to be 27.9 ± 1.8 nm (*n* = 5). Zeta potential analysis confirmed that all the metallic NMs were negatively charged in water (Figure 1C, Table 2).

### 3.2. The Effect of Sublethal Concentrations of Metal Nanomaterials on Cell Activity

**Figure 2:**
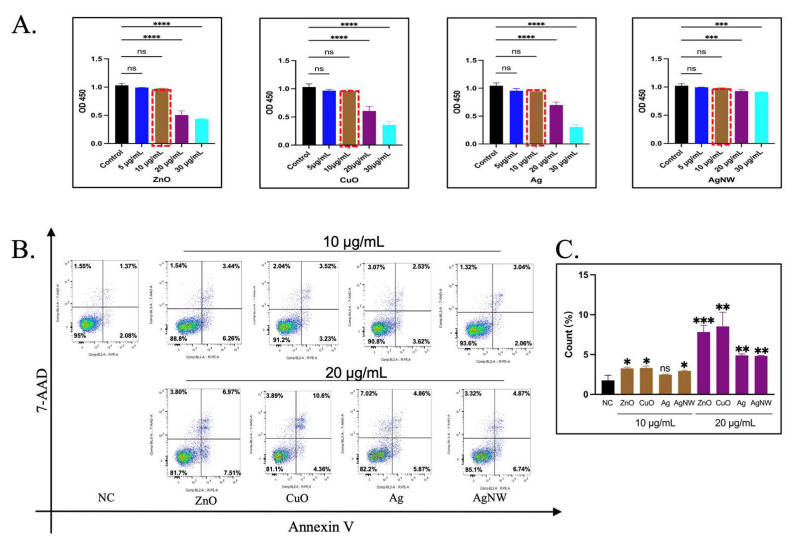
Cytotoxicity and apoptosis detection in HK-2 cells treated with the four types of metallic nanomaterials. (**A**) Cell cytotoxicity was tested using CCK8 assay. HK-2 cells were treated with ZnO, CuO, Ag, and AgNW at 5, 10, 15, and 20 μg/mL for 24 h, respectively. The data were presented as a mean ± SEM of three independent experiments. * *p* < 0.05, ** *p* < 0.01, *** *p* < 0.001, **** *p* < 0.0001, ns > 0.05 with respect to the control based on one-way ANOVA. (**B**) Representative FACA scatter plots of apoptosis for HK-2 cells were treated with 10 μg/mL of ZnO, CuO, Ag, and AgNW relative to untreated HK-2 cells using Annexin V-PE staining. The control group was treated with 20 μg/mL of NMs. Experiments were performed in biological triplicates. (**C**) Statistical analysis of the percentage of apoptotic cells in HK-2 cells treated with different nanomaterials concentrations. Averaged data from Annexin V-FITC assay showing the ability to induce apoptosis. * *p* < 0.05, ** *p* < 0.01, *** *p* < 0.001 vs. NC.

CCK8 assay is a rapid and sensitive cytotoxicity assay that relies on the detection of dehydrogenase activity in viable cells. HK-2 cells were treated with the four types of metal NMs at 5, 10, 20, 30 μg/mL for 24 h. As shown in Figure 2A, the prepared metal NMs exhibited different cytotoxicity profiles. Compared with untreated cells, HK-2 cells showed no significant cytotoxicity after treatment with ZnO, CuO, Ag, and AgNWs at 5 μg/mL and 10 μg/mL for 24 h. An increase in the concentration of the three kinds of NMs caused significant cell toxicity at 20 μg/mL. The survival rate of the cells reached 50–70%, and with an increase in the concentration, the cytotoxicity became more obvious at 30 μg/mL, while the survival rate of AgNMs at 20 and 30 μg/mL reached 30%. Compared with the concentration of 10 μg/mL, there was a significant toxic death. The reason for these results may be that the cytotoxicity of metallic NMs depends on their duration of exposure, dosage, and concentration, as well as the types of cells [34]. Based on the above results, a critical concentration of 10 μg/mL was selected as the sublethal NMs concentration for HK-2 cells. To confirm this finding, apoptotic staining was performed on HK-2 cells stimulated with the four types of metallic NMs (Figure 2B). Flow cytometry analysis showed mild apoptosis of HK-2 cells compared to untreated cells after 24 h of treatment at an NM concentration of 10 μg/mL (1.37% (Control) vs. 3.44% (ZnO) vs. 3.52% (CuO) vs. 2.53% (Ag) vs. 3.04% (AgNW) for Annexin V+PI+ cells, while apoptosis was noted at a concentration of 20 μg/mL, which was consistent with the cytotoxicity assessment (1.37% (Control) vs. 6.97% (ZnO) vs. 10.6% (CuO) vs. 4.86% (Ag) vs. 4.87% (AgNW) for Annexin V+PI+ cells. 20 μg/mL of metallic nanomaterials increased the percentage of apoptosis compared with 10 μg/mL (Figure 2C). Thus, 10 μg/mL was selected as the sublethal compression concentration for HK-2 cells.

### 3.3. The Effects of Metallic Nanomaterials on the Mitochondria in HK-2 Cells

**Figure 3:**
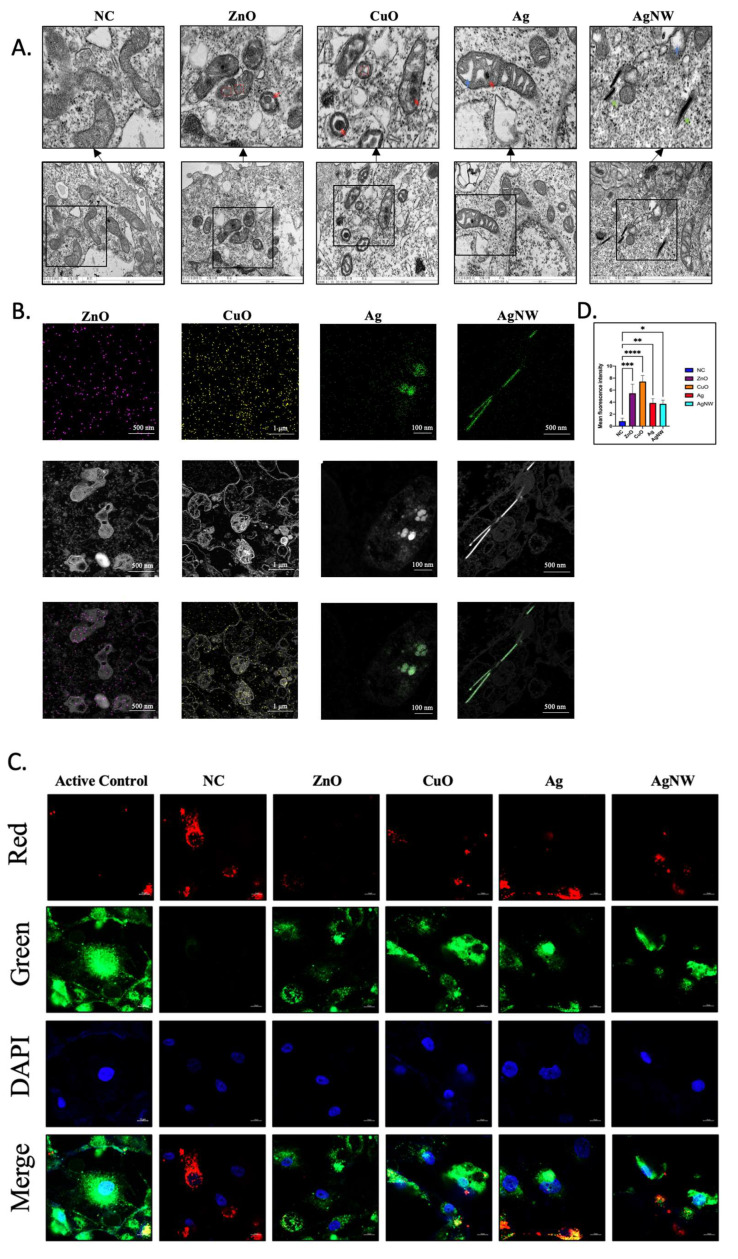
Characterization of mitochondrial structure. (**A**) TEM images of mitochondria of HK-2 cells treated with or without ZnO, CuO, Ag, and AgNW. The original magnification is 60,000×. (**B**) TEM mapping images and corresponding elemental mappings of Zn (purple), Cu (yellow), Ag (green). (**C**) Confocal microscopy images of HK-2 cells with JC-1 staining for mitochondrial membrane potential analysis. Scale bar is 10 μm. (**D**) The quantification of fluorescent intensity in (**C**). Ratios of two colors (green/red) in cells exposed to the four types of metallic nanomaterial treatment was calculated by a microplate reader (*n* = 3). * *p* < 0.05, ** *p* < 0.01, *** *p* < 0.001, **** *p* < 0.0001 with respect to the control based on one-way ANOVA.

Studies have shown that metallic NMs may cause mitochondrial dysfunction, leading to morphological changes, increased reactive oxygen species (ROS) production, decreased mitochondrial membrane potential, and inhibition of enzyme activities, electron transport chains, and cellular respiration, leading to cell apoptosis and necrosis [35,36,37]. We explored whether the damage caused by metal NMs was reflected in organelles at sublethal concentrations before cell death. Mitochondria, which are the organelles most sensitive to external changes, were the key focus of our research. First, we investigated whether sublethal concentrations of metallic NMs caused mitochondrial structural changes using TEM imaging (Figure 3A). After 24 h of exposure to metallic NMs, treated HK-2 cells showed that the matrix of mitochondria formed spheres in the ZnO- and CuO-treated groups (marked by a red box) and formed partial cavities in the Ag- and AgNW-treated groups (blue arrow), which could be because of cristolysis and a lack of cristae. Similar results have previously shown that metal NMs can induce a series of morphological changes in the mitochondria of mouse liver cells [30]; however, the effect of sublethal concentrations on the mitochondrial structure in HK-2 cells is unclear. In addition, treated HK-2 cells showed mitochondrial membrane shrinkage in all treatment groups. Surprisingly, the ZnO, CuO, and AgNMs were also detected in the mitochondria (red arrow), and the AgNWs were observed in the cytoplasmic matrix (green arrow). NMs enter cells and organelles and affect their functions. We have performed an energy spectrum test. As revealed by transmission electron microscope, ZnO, CuO, and Ag nanomaterials experimental groups, respectively detected the clear distribution of Zn, Cu, and Ag signals in mitochondrial organelles, demonstrating that metal nanomaterials enter mitochondria under sublethal concentration. It is worth noting that AgNWs are still located in the cytoplasm. It might have something to do with its length (Figure 3B). JC-1 was used to stain the mitochondria to indicate mitochondrial membrane potential. Thus, a shift in fluorescence from red to green indicates a decrease in the mitochondrial membrane potential. After 24 h of exposure, we evaluated the mitochondrial membrane potential using confocal microscopy (Figure 3B). HK-2 cells treated with metallic NMs at a concentration of 10 μg/mL showed an increase in green fluorescence compared with untreated HK-2 cells, especially HK-2 cells exposed to ZnO and CuO. The ratio between green and red fluorescence also showed that 10 μg/mL of ZnO and CuO had a greater influence on the mitochondrial membrane potential than the other concentrations (Figure 3C). Overall, our findings indicated that sublethal doses of 10 μg/mL were able to produce mitochondrial damage in HK-2 cells leading to minor levels of cell death. In addition, HK-2 cells treated with 10 μg/mL of ZnO and CuO cause stronger mitochondrial damage than Ag and AgNW treatments.

### 3.4. The Effects of Metallic Nanomaterials on the Mitochondrial Energy Metabolism

**Figure 4:**
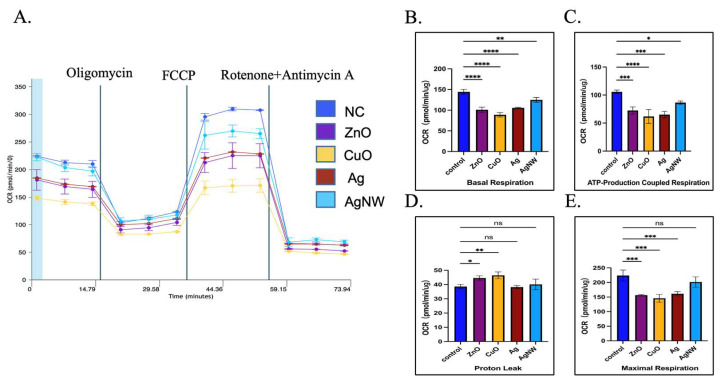
Mitochondrial energy metabolism assay. (**A**) Bioenergetic profiles of HK-2 cells treated with 10 μg/mL of ZnO, CuO, Ag, and AgNW measured by Seahorse XF96. (**B**–**E**) Mitochondrial OXPHS were analyzed based on basal respiration, ATP production, proton leak, and maximal respiration for HK-2 cells treated with ZnO, CuO, Ag, and AgNW nanomaterials. * *p* < 0.05, ** *p* < 0.01, *** *p* < 0.001, **** *p* < 0.0001, ns > 0.05 with respect to control based on one-way ANOVA.

The major function of mitochondria is to supply ATP for cellular energy metabolism [38,39]. We found that treatment of HK-2 cells with metal NMs caused changes in mitochondrial structure and mitochondrial damage. To assess whether mitochondrial damage caused by 10 μg/mL of the four types of metallic NMs causes changes in cell energy metabolism, we performed a seahorse mitochondrial pressure assay to assess mitochondrial function by real-time monitoring of the oxygen consumption rate (OCR). Figure 4A shows the impact of oligomycin, FCCP, and a mixture of rotenone and antimycin A on the OCR curve obtained using treated and untreated HK-2 cells. We found that untreated HK-2 cells had higher basal respiration than treated HK-2 cells (*p* **** < 0.0001 for ZnO, *p* **** < 0.0001 for CuO, *p* **** < 0.0001 for Ag, *p* ** = 0.0046 for AgNWs), indicating a much higher endogenous ATP requirement in untreated HK-2 cells (Figure 4B), and the energy demand of metal NM-treated HK-2 cells was inhibited under basic conditions.

Oligomycin is an inhibitor of ATP synthase that reduces electron flow through the electron transport chain [22]. After oligomycin addition in the treated HK-2 cells, mitochondrial respiration reduction was observed, based on a decline in OCR linked to ATP production. Figure 4C shows that the oxygen consumption used to produce mitochondrial ATP was lower in treated HK-2 cells than in untreated HK-2 cells (*p* *** = 0.0006 for ZnO, *p* **** < 0.0001 for CuO, *p* *** = 0.0001 for Ag, and *p* * = 0.0227 for AgNW). We also tested for proton leakage (Figure 4D). HK-2 cells treated with 10 μg/mL of ZnO and CuO showed differences in proton leakage (*p* * = 0.0282 for ZnO and *p* ** = 0.0056 for CuO), while there was no difference in the Ag- and AgNW-treated groups when compared with untreated HK-2 cells.

HK-2 and untreated HK-2 cells were treated with FCCP (Figure 4E). FCCP is a strong uncoupler of oxidative phosphorylation to prevent proton flux in the mitochondrial inner membrane leading to proton gradient damage and mitochondrial membrane potential disruption [40,41]. After the addition of FCCP, the treatment of HK-2 cells with these metallic nanoparticles resulted in a significant increase in the OCR after AgNW treatment compared to that in untreated HK-2 cells (*p* *** = 0.0004 for ZnO, *p* *** = 0.0001 for CuO, *p* *** = 0.0007 for Ag, and *p* = 0.2004 for AgNW), suggesting that these metallic NMs disrupt the electrochemical gradient of the electron transport chain of HK-2 cells, resulting in the inability of ATP synthase to synthesize ATP. This also reflects the reduced ability of treated HK-2 cells to use membrane potential, which was similar to the mitochondrial membrane potential of treated HK-2 cells described in Section 3.3. The aerobic respiration of HK-2 cells was inhibited by a sublethal concentration of metal NMs, and the ability of the cells to react to increased energy demand was weakened. The ability of HK-2 cells treated with metal NMs to consume oxygen to drive the cell to break down the nutrient matrix and release energy to produce large amounts of ATP is reduced, and the function of mitochondria as the cell’s “energy factory” is inhibited. Inhibition of mitochondrial metabolism may be the key to late cell death.

### 3.5. The Effects of Metallic Nanomaterials on the Mitochondrial Complex Activity

**Figure 5:**
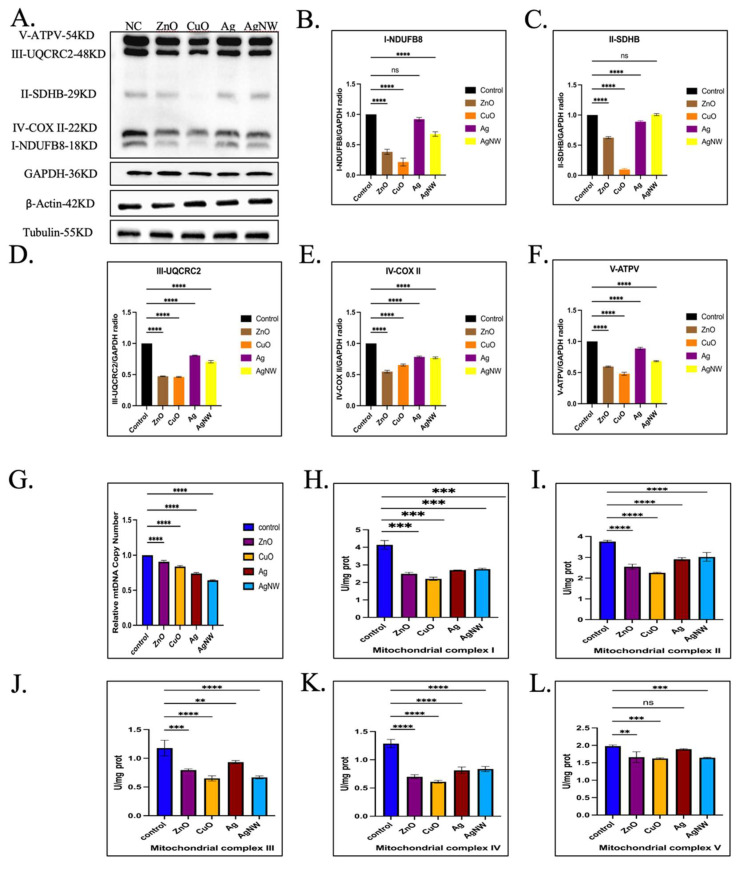
Activities of mitochondrial complexes assay. (**A**) Western blot analysis of HK-2 cells upon exposure to 10 μg/mL of the four types of metallic nanomaterials for 24 h. (**B**–**F**) The band density ratio of NDUFB8, SDHB, UQCRC2 COXII, and ATPV to GAPDH in the Western blots was quantified by densitometry. (**G**) Analysis of mtDNA expression for HK-2 cells exposed to metallic nanomaterials for 24 h (*n* = 3). (**H**–**L**) The activity of the mitochondrial respiration chain complexes I to V was tested using a detection kit after the HK-2 cells were treated with the four types of metallic nanomaterials for 24 h. The NADH oxidation rate was measured at 340 nm using the plate reader. ** *p* < 0.01, *** *p* < 0.001, **** *p* < 0.0001, ns > 0.05 with respect to the control based on one-way ANOVA.

We found that basic respiration, ATP production, and maximum respiration of mitochondria were affected by treatment with metallic NMs for HK-2 cells in Section 3.4. The electron transport chain located in the inner mitochondrial membrane correlates with these parameters. The mitochondrial electron transport chain, including mitochondrial respiratory chain complexes (I–IV), together with F1F0-ATP synthase (complex V), form the basis of ATP production during oxidative phosphorylation [42,43]. We first examined the protein expression of the mitochondrial respiratory chain complex (I–V) in HK-2 cells treated with the four metallic NMs (Figure 5A–F). The results showed that, when compared to untreated HK-2 cells, treatments with the four types of metallic NMs had obvious inhibitory effects on the related proteins of mitochondrial complexes I, III, IV, and V, especially ZnO and CuO, which were particularly effective. Notably, the AgNWs did not affect the expression of mitochondrial complex II-related proteins (Figure 5C). Next, we detected the expression level of mtDNA in HK-2 cells after the addition of four types of metallic NMs, based on evidence that mitochondrial DNA (mtDNA) encodes many proteins essential for the assembly and activity of the mitochondrial respiratory complex [42,43]. As shown in Figure 5G, all metallic NMs inhibited mitochondrial mtDNA (*p* = 0.0011 for ZnO, *p* **** < 0.0001 for CuO, *p* **** < 0.0001 for Ag, and *p* **** < 0.0001 for AgNWs). Next, the activities of mitochondrial complexes were assessed (Figure 5H–L), and the results showed that the activities of mitochondrial respiratory chain complexes II, IV, and V were affected by CuO treatment in HK-2 cells compared to untreated HK-2 cells, followed by ZnO treatment. In addition, the four metallic NMs had the greatest influence on the activity of mitochondrial complex IV and the least influence on the activity of mitochondrial complex III. It is believed that metal NMs with sublethal concentrations enter the mitochondria and impact the mitochondrial complex, affecting its activity and expression, thus affecting mitochondrial energy metabolism.

## 4. Conclusions

ZnO, CuO, and Ag nanoparticles aggregated in the mitochondria, and AgNW entered the cytoplasmic matrix of HK-2 cells when treated with a sublethal concentration of metal NMs. The entry of metal nanoparticles into mitochondria can affect the mitochondrial structure and function of HK-2 cells, causing significant damage to the mitochondria and inhibiting energy metabolism. It not only greatly inhibits the activity of the mitochondrial complex but also affects the expression of the mitochondrial complex. In addition, the mitochondrial damage caused by ZnO and CuO was significantly higher than that caused by Ag and AgNW NMs, which may be related to the physical and chemical properties of the metallic materials and is worthy of further exploration. It is worth noting that this sublethal concentration had little effect on cytotoxicity in the short term, which provides a better understanding of organelle damage before cell death. Therefore, in the future, we will focus on the long-term stimulatory effects of sublethal concentrations of NMs on cells and explore the dynamic changes in organelles and cells under long-term stimulation conditions.

## Figures and Tables

**Table 1:** List of primers used in the study.

Primers	Sequence
D-Loop F	GGCTCTCAACTCCAGCATGT
D-Loop R	AGGACGAGGGAGGCTACAAT
G6PC F	CTGTCTTTGATTCCTGCCTCAT
G6PC R	GTGGCTGTGCAGACATTCAA

**Table 2:** Characterization of the four types of metallic nanomaterials.

Nanomaterials	Size ^1^	Size ^2^	Zeta Potential
ZnO	40–150 nm (TEM)	129.5 ± 38.7 nm	−2.2 ± 0.6
CuO	10–50 nm (SEM)	50.1 ± 10.9 nm	−29.6 ± 0.8
Ag	30 nm (TEM)	30.3 ± 0.4 nm	−31.9 ± 3.0
AgNW	30 nm (SEM)	245.1 ± 1.5 nm	−7.3 ± 1.8

^1^ The sizes of the four types of nanomaterials were calculated by TEM and SEM from the supplier (XFNANO). ^2^ The sizes of the four types of nanomaterials were detected by DLS.

## Data Availability

Not applicable.
